# Clinician Decision-Making Around Offering Home Video Telehealth Visits: Qualitative Study

**DOI:** 10.2196/83800

**Published:** 2026-06-18

**Authors:** Megan E Gately, Steven D Shirk, Emma D Quach, Lauren R Moo

**Affiliations:** 1 New England Geriatric Research Education and Clinical Center VA Bedford Healthcare System Bedford, MA United States; 2 Division of Geriatrics Boston University Chobanian & Avedisian School of Medicine Boston, MA United States; 3 Center for Health Optimization and Implementation Research VA Bedford Healthcare System Bedford, MA United States; 4 Department of Public Health University of Massachusetts Lowell Lowell, MA United States; 5 Department of Neurology Harvard Medical School Boston, MA United States

**Keywords:** telemedicine, telehealth, older adult, health services for the aged, geriatrics, veterans health

## Abstract

**Background:**

Use of in-home video telehealth rapidly expanded in response to the COVID-19 pandemic, including at Veterans Affairs (VA), a forerunner in telehealth. Despite this uptick, differences in use by patient age and rurality created a digital divide that persists to this day. While clinicians frequently cite patients’ older age and lack of technical skills as barriers to in-home video telehealth, it remains unclear how clinicians decide whether to offer video visits to patients and to what extent these beliefs may hinder offering video visits to older adults. Gathering perspectives from clinician users of in-home video telehealth may illuminate opportunities to ensure continued access to care through solutions such as telehealth.

**Objective:**

This study aimed to examine clinician decision-making around the offer of in-home video telehealth to understand how interprofessional clinicians determine to whom they offer in-home video telehealth and what factors (organizational, personal, or attitudinal) influence their decision.

**Methods:**

We conducted a qualitative study by using semistructured interviews. Participants were interprofessional clinicians (N=16) employed by 11 different VA hospitals and included 1 clinical pharmacist, 6 medical doctors, 2 nurse practitioners, 1 occupational therapist, 3 psychologists, 1 physical therapist, 1 speech-language pathologist, and 1 social worker. All the participants had at least some experience using in-home video telehealth from locations across VA (the largest integrated health care system in the United States) and were interviewed over a 6-month period. Interviews focused on clinicians’ use of video telehealth and the decision-making process involved in offering in-home video telehealth. We used directed content analysis with a rapid analytic approach, given the time-pressured nature of our project.

**Results:**

This study revealed that clinician decision-making around offering in-home video visits is complex and influenced by several domains, namely, (1) clinician factors, including experience with video and perceived benefits of video; (2) appointment factors, including the visit’s clinical goal; (3) clinician-reported patient factors, including age and willingness to try video; (4) patient social context, including caregiver availability; (5) geographical factors, such as availability of reliable high-speed internet and patient distance from the medical center; and (6) health system factors, including technical support and clinician ability to work from home. Access to in-home video telehealth may be facilitated by clinician familiarity and confidence with telehealth technology, strategies to improve patients’ technical skills, and support for caregivers. Infrastructure also plays an important role, including device availability, broadband reliability, and clear protocols for matching services to video visits.

**Conclusions:**

Findings highlight the importance of clinician competency in telehealth, patient and caregiver digital readiness, and a supportive technology infrastructure to equitable in-home video care. In addition, improved guidance for specific clinical services and awareness of potential biases may enable consistent, accessible telehealth delivery for older adults and medically complex populations.

## Introduction

Rapid uptake of in-home video telehealth during the COVID-19 pandemic accelerated the Veterans Affairs (VA) goal to increase patient access to care using telehealth technologies [1-5]. While VA, the largest single health care provider in the United States, had for decades offered telehealth in various forms—telephone, clinic-to-clinic video telehealth, and store-and-forward services [6]—in-home video telehealth (a live, synchronous encounter) was used to a lesser degree prior to COVID-19. Despite an increase in use of in-home video telehealth during the COVID-19 pandemic, age-related differences emerged, with analyses of VA outpatient encounters showing that veterans aged ≥65 years used video-based care at lower rates than younger veterans [1] and that older veterans tended to prefer telephone to video [7]. Extant literature further highlighted that older high-need, and high-risk veterans, such as those with frailty, face significant barriers to adopting video, including cognitive challenges and lack of technological resources [8]. Although the COVID-19 pandemic prompted a substantial shift toward virtual care, age-related disparities in technology use were only temporarily reduced, not eliminated.

Older adults generally use technology less than younger adults [9], a trend that continued even during the COVID-19 pandemic as demonstrated by lower rates of video telehealth use [10-13] and lower use of mobile health technology more broadly among older adults [14]. However, some evidence suggests that the gap in technology use between younger and older adults is narrowing [15]. Regardless, the urgency of the COVID-19 pandemic undoubtedly led clinicians to offer video telehealth to patients who were not previously considered ideal candidates, including older adults. Certain health and demographic factors may constrain older adults’ ability to engage with video, such as complex medical needs [16,17] and sensory impairments such as vision and hearing loss [18,19]. However, such challenges make video telehealth an attractive option for older adults as they may operate as barriers to in-person care. Additional care access challenges are more common among the veteran population, such as rurality, unstable housing, and lower socioeconomic status [20,21], underscoring the importance of telehealth to meet care needs. In fact, internet access (a prerequisite for video telehealth) is increasingly recognized as a “super determinant of health” [22], reflecting a recognition of the link between access to telehealth and overall health and wellness.

Clinician decision-making may be a critical factor in ensuring equitable access to video telehealth by patients with complex care needs, including older adults. Prior studies of video telehealth use among older adults served within and outside VA suggest various clinician factors associated with video telehealth use but leave important knowledge gaps. While clinician decision-making around the offer of video telehealth to older patients is understudied, older age and lack of technical skills are barriers frequently cited by clinicians [23-25], suggesting an ageist bias against older adults. A large nationwide (non-VA) survey of doctors, nurses, and other clinicians revealed that most clinicians believed telehealth is “dangerous” or an unrealistic option for older adults [26]. Evidence regarding VA clinicians’ views on telehealth identifies limited veteran technical skills as a barrier [27], but it remains unclear how clinicians assess veteran skill level or readiness for video telehealth. One qualitative study observed that VA clinicians assumed older adults did not want or would not accept video [28]. However, we do not know the range of factors, for example, appointment type, clinic focus, or health care system factors, that clinicians consider when deciding whether to offer telehealth.

Given this knowledge gap, we conducted a study of clinicians to examine factors that influence the offer of video telehealth. The multiple factors clinicians consider when deciding whether to make an initial offer of in-home video telehealth may identify opportunities for intervention to promote TechQuity [29]. TechQuity refers to the intentional use of technology in health care to promote equity. To ensure equitable distribution of video telehealth to patients who need it the most, it is critical to identify barriers and facilitating factors. Specifically, this study aimed to understand how interprofessional clinicians determine to whom they offer in-home video telehealth and what factors (organizational, personal, or attitudinal) influence their decision.

## Methods

### Study Design

We conducted a qualitative study using semistructured interviews. We used rapid analytic techniques as this study was part of a 1-year funded program evaluation. To increase methodological rigor, we used the 18-item Planning for and Assessing Rigor in Rapid Qualitative Analysis framework [30] (refer to Multimedia Appendix 1 for the completed Planning for and Assessing Rigor in Rapid Qualitative Analysis checklist).

### Study Team

The study team included 4 VA researchers with expertise in program evaluation, qualitative methods, and geriatrics care (including use of telehealth). The team was led by a clinician researcher (MG) with more than 10 years of experience leading clinical and research projects involving older adults and telehealth and clinical and health services researchers (LM, SS, and EQ) with expertise in geriatrics and gerontology, social psychology, telehealth, and qualitative methods. Our broad-based expertise (including diverse professional backgrounds) strengthened the rigor of the analysis by supporting triangulation, reducing the influence of any single researcher’s biases, and enabling more comprehensive interpretation of interview data. We also used structured reflexive practices, including regular team-based discussions and collaborative analysis, to systematically monitor how our prior knowledge and professional positioning might introduce bias into the rapid appraisal process. All study team members collaborated on the study design, data collection, and analysis, which ensured resulting factors accurately represented participant experiences rather than preconceived assumptions or the constraints of expedited data collection.

### Participant Recruitment

To examine clinicians’ decision-making around offering in-home video, we invited interprofessional VA clinicians with expertise in geriatrics video telehealth to participate in brief semistructured interviews. There were no other inclusion criteria. Our sampling method, including eligibility criteria and recruitment strategies, was determined prior to inviting clinicians to participate. Clinicians were identified by study team members, in part by leveraging our relationship with GRECC-Connect, a national network of geriatrics video telehealth experts at VA [31,32]. Clinicians were invited to participate via an email from a member of the study team, with all 16 invited clinicians agreeing to participate. Participants were employed by 11 different VA hospitals and included 1 clinical pharmacist, 6 medical doctors (specialists in endocrinology, family medicine, geriatrics, primary care, and psychiatry), 2 nurse practitioners, 1 occupational therapist, 3 psychologists, 1 physical therapist, 1 speech-language pathologist, and 1 social worker. Other than role and VA location, no other demographic details were collected.

### Data Collection

Prior to conducting interviews, we established data collection and documentation processes to ensure uniform practices, including identifying secure storage locations and interview procedures. Interview questions were derived from a focused interview guide that we developed and piloted internally within our team and iteratively refined based on team input. Questions addressed clinicians’ in-home video experience and decision-making regarding the initial offer of video. Interviews were conducted between January and June of 2024. Interviews lasted 26 minutes on average (SD 5.79; range 16-37 minutes) and were led by a study team member with a second team member observing, taking notes, and asking clarifying questions. Interviews were recorded in Microsoft Teams, which autogenerated interview transcripts to be used for analysis. Transcripts were cross-checked with audio recordings prior to analysis to ensure accuracy.

### Data Analysis

We conducted a directed content analysis [33] using a rapid analytic approach [34,35], given our project’s compressed 1-year timeline. Analysis focused on identifying factors influencing clinicians’ decisions to offer in-home video telehealth. To support analysis and promote consistent summarization, we (MG, LM, SS, and EQ) used working documents developed by the Rapid Research, Evaluation and Appraisal Lab (RREAL) that facilitate data synthesis while data collection is ongoing, that is, RREAL sheets [35]. These are structured, thematic templates that allow for real-time data transfer. To organize thematic data, we developed RREAL sheet summary templates aligned with the study’s analytic domains and interview questions, organized in Microsoft Word. RREAL sheets were structured to summarize key findings, quotes, and observations related to factors found to influence clinician decision-making. We then developed explicit rules for populating data into RREAL sheets, with analysis starting before all interviews were completed. Team members first individually completed RREAL sheets for each interview, with each transcript being reviewed by at least 2 members of the study team who were not present during the interviews. Then, during regular team meetings, we organized data into a consensus RREAL sheet in which data in each analytic domain were synthesized across participants, ensuring version control. We used constant comparison to identify patterns and variations across the dataset until saturation [33], the point at which no new information emerged and we reached consensus on the final list of factors.

### Ethics Approval

In accordance with institutional procedures, this project was reviewed by the institutional review board of VA Bedford Healthcare System. Ethics approval was waived, as the project was determined to be a nonresearch program evaluation of existing VA clinical services. Though the project was determined to be nonresearch, all study procedures were conducted in adherence with VA ethical and privacy protections and ethical standards consistent with the revised (2000) Helsinki Declaration. Specifically, though no formal consent was required, we informed participants about study objectives, that they could decline to participate at any time, that data would be kept secure and confidential, and that they could choose not to be recorded. Participants were not compensated for participation, in accordance with VA policy regarding employees.

## Results

### Overview

Analysis of the semistructured interviews (N=16) revealed key factors influential to clinician decision-making around the offer of in-home video visits related to the clinician, appointment, patient, patient social context, geography, and health system. Six factors were identified: (1) clinician factors, (2) appointment factors, (3) clinician-reported patient factors, (4) patient social context, (5) geographical factors, and (6) health system factors, each of which influenced decision-making to varying degrees. Descriptions and examples for each are provided in the following sections.

### Clinician Factors

Factors influencing clinician decision-making included their experience with video and perceived benefits of video. As noted above, all participants had experience using in-home video telehealth in their practice with older adults, but experience levels varied. One nurse practitioner, a self-described video “champion,” noted that, over 7 years ago, her team was “one of the first in the country to do [video] towards geriatrics” (participant #4). Another, an endocrinologist, described herself as a “late adopter” of video, describing how she began conducting telephone visits during COVID-19 and then “finally...learned how to do” video after recognizing the limitations of phone visits (participant #7). Some discussed how their commitment to video influenced older adults’ ability to successfully engage, as when a psychologist discussed trying to “hang in” while assisting an older adult, stating, “once I get set up, they are thrilled, excited, possibilities open up for them” (participant #16). While COVID-19 was often the catalyst for in-home video visits, most clinicians saw benefit in their continued use after the pandemic. Enhanced clinical assessment was a perceived benefit, with 1 nurse practitioner noting that she gets “a better sense” of patients’ needs over in-home video versus in the clinic (participant #13). Interviewees reported that their colleagues’ decision-making regarding video visits often differed from their own, with colleagues either having lower technological comfort or believing care cannot be translated to video. A geriatrician stated that her coworkers “are more impatient...[with] not as much grit,” especially around troubleshooting patients’ technology problems (participant #12).

### Appointment Factors

Appointment purpose and care type influenced clinicians’ decision to offer in-home video. Clinicians generally used in-home video for wide-ranging clinical services, including rehabilitation, clinical pharmacy, social work, psychiatry, and primary care, often in combination with other forms of telehealth, for example, telephone. Video appointments also optimized certain services, such as medication management and home safety assessments, by allowing observation of patients’ routines and home environments. A clinical pharmacist described video as a great option for medication reconciliation, as patients “don’t have to bring all their medication bottles in” (participant #14). While several clinicians conducted all visits via in-home video during the pandemic, many described a more nuanced approach after the pandemic. For example, a physical therapist assessing older patients’ mobility shifted from conducting initial assessments in patients’ homes during COVID-19 to conducting them via clinic-to-clinic telehealth, in which the patient is at a satellite VA clinic and assisted by a telehealth technician, after clinics reopened (participant #3). Some clinicians noted limitations of in-home video for certain diagnostics (eg, urinalysis) and testing. One neuropsychologist described patient difficulty accessing the full screen feature during cognitive testing (participant #2), which influenced test administration. Medical acuity was also a factor in offering in-home video, but the influence on video varied by clinician; some felt in-home video was an expeditious way to triage acute issues, whereas others felt acute issues necessitated an in-clinic visit.

### Clinician-Reported Patient Factors

Patient preference, age, medical complexity, and technical literacy also influenced whether clinicians offered in-home video. According to clinicians, some patients prefer in-person clinic visits, particularly those who are hesitant about technology or who value the social connection associated with attending clinic visits. One psychiatrist noted that a lot of his older patients prefer to come in person, as “They’re not so good with the technology” (participant #5). In a similar way, a speech-language pathologist described that after she decides whether the type of care can be translated to video, “I’m then starting to ask myself or the patient [sic] their level of comfort with technology” (participant #11). Several clinicians described lived experience with older patients having difficulty with logging in and effective camera management (eg, challenges getting angles right). Older age alone was rarely mentioned as influential, with 1 physician noting, “You think younger people are going to be better [at telehealth], but my parents are better at computers than I am” (participant #15). Though not explicitly referring to age, some clinicians mentioned common age-related challenges, such as impaired cognition and hearing loss, which can be exacerbated during video visits. One physician stated, “hearing problems are sort of compounded by a telehealth format” (participant #1). For others, however, “age is definitely a factor,” with 1 physician stating that she was “more likely to offer it [video] to young patients” (participant #6).

### Patient Social Context

Availability of caregivers frequently influenced the likelihood that clinicians offer in-home video, particularly for patients with impaired cognition. One physician noted several patients “probably would not have been able to meet with me without a caregiver to set them up” (participant #1). Older patients reportedly often need caregiver assistance, specifically with logging in and device operation. Caregivers were particularly helpful for patients with cognitive impairment, as when a speech-language pathologist noted that patients with dementia “required some level of support in the home like a caregiver” (participant #11). The fact that video enabled caregiver participation, including contributing to discussions and receiving patient education, positively influenced clinicians’ willingness to offer in-home video visits. A geriatrician noted that caregivers “want to be involved and they’re making the decisions and kind of managing the care” (participant #15). In contrast, absence of a caregiver limited offers of video. As an alternative to family caregivers, some clinicians described collaborating with home-based clinicians to facilitate patient access to in-home video, with 1 physician noting, “it’s probably a nurse that you’re underutilizing when you’re thinking about video follow-up” (participant #15). Though perceived as less integral for certain types of care (eg, psychiatry), caregivers were generally considered a decisive factor due to their ability to assist with technology. A noted benefit of in-home video telehealth was increased convenience for working caregivers.

### Geographical Factors

Greater patient distance increased the likelihood of offering in-home video visits, with many clinicians describing how video enabled access for rural patients who otherwise travel long distances for in-person care. This was particularly true for clinicians unable to travel to patients’ homes, with 1 home-based primary care nurse practitioner stating, “not everybody has the luxury that I have to hop in a car and just go in to see them in their house” (participant #13). Relatedly, 1 social worker described patients’ “transportation issues” (participant #8) as limiting their ability to travel to care. For older patients, any distance was perceived as a barrier, with several clinicians mentioning mobility and other challenges common to older adults (eg, cognitive impairment) as significant obstacles. A psychiatrist related that video was helpful “if they’re having trouble with the logistics of getting here, like, they’re on a wheelchair or cane” (participant #5). In contrast, rural patients’ inability to access reliable high-speed internet sufficient for in-home video visits, due to infrastructure challenges, constrained the offer of video.

### Health System Factors

System-level factors influencing the offer of in-home video included clinicians’ ability to telework, availability of technology and technical support, and scheduling processes. Scheduling was the primary system-level barrier to video. Several clinicians described limitations in scheduling workflows, with 1 physician noting, “the system favors face-to-face” (participant #7). The ability to telework positively influenced the use of in-home video visits, particularly for clinicians who provided care exclusively via video, such as a psychiatrist serving rural areas who sees patients only remotely. A psychologist also described the ability to provide video visits while out with an injury, stating, “I have like a foot injury, so right now it’s [video] 100% of my time” (participant #10). Video was also seen as a flexible, convenient option for clinicians working from home due to clinic space limitations. Influential technological factors included the availability of VA-issued tablets for patients who lack their own, VA’s proprietary HIPAA (Health Insurance Portability and Accountability Act)–compliant videoconferencing software, and test calls. A physician described that if a veteran lacked a videoconferencing device, “I would offer to do a Digital Divide consult” (participant #1) to provide them with a VA-issued tablet.

## Discussion

### Principal Findings

The findings of this study demonstrate that clinician decision-making around the offer of in‑home video telehealth is shaped by myriad factors spanning clinicians, appointments, patients, social context, geography, and health systems. Key findings include clinicians’ prior experience and perceived clinical value of video and appointment type as influential factors. Patient preferences, cognitive or sensory limitations, and technological literacy were also driving factors in the offer of video and were often intertwined with the availability of caregivers who facilitated patient access to video-based care. Geographic distance and transportation barriers increased the likelihood of offering video, whereas limited internet access in rural areas constrained its use. Finally, system-level elements, including scheduling workflows, telework flexibility, and the availability of devices and technical support, either enabled or impeded clinicians’ ability to integrate in-home video into routine care. In the following sections, we discuss the impact of each factor in the context of extant literature.

### Clinician and Appointment Factors

The finding that clinicians’ prior use of video can either facilitate or detract from adoption aligns with evidence from various disciplines [36-38], suggesting that increased clinician familiarity with video telehealth technology may increase their willingness to offer in-home video to patients. Our prior work surveying VA occupational therapy practitioners found that participants using video telehealth expressed greater comfort with video telehealth to deliver occupational therapy services [37]. Similarly, our finding that clinicians perceive benefits in seeing patients in their homes echoes our prior work in which we used video for home safety assessments and dementia management [37,39-41]. Using video to address home safety may be of particular salience for older adults, given that falls are a key predictor of older adult injury and disability [42]. Similarly, our finding that video may facilitate medication management aligns with other work [39,43], streamlining clinicians’ ability to ascertain patients’ home medication routines. These findings have potential relevance for other disciplines that involve visualizing patients in their natural context, including primary care, nursing, occupational therapy, and rehabilitation. For example, a study of telerehabilitation with pediatric patients reported that delivering exercise programs via telehealth in the home enhanced caregiver training outcomes [44].

Also related to appointment factors, our study connects to a small but growing literature on how clinicians match clinical services to specific forms of telehealth. For instance, our finding that some clinicians prefer video for triaging acute patient issues aligns with studies from primary care [45]. Perceptions that video has limitations for certain services, such as physical examination, have also been observed. One study observed how patient factors, including decreased mobility and associated safety risks, may complicate video-based physical examination, while highlighting the potential for adjunctive forms of technology, such as wearable sensors, to help mitigate these challenges in the future [46]. Another study highlighted how internet bandwidth may undermine the accuracy of physical function measurements, possibly leading to misdiagnosis [47]. Similarly, a recent study examining teleaudiology in a pediatric population highlighted the risk of error in teleassessments, suggesting that more experienced clinicians could train novice clinicians [48]. Taken together, these findings highlight the need for further research on how specific types of clinical services influence clinicians’ service modality choice and for more detailed guidance on how to conduct certain types of clinical care via in-home video.

### Clinician-Reported Patient Factors and Social Context

Regarding clinician-reported patient factors, our finding that patient preference influences clinicians’ offer of video aligns with extant literature [49]. This underscores the importance of integrating in-home video as a care delivery format option where possible while balancing patient priorities. Our finding that patient hesitancy toward video may be linked to self-described low technical capacity suggests the need to assess patient technical literacy. Tools such as the recently developed Digital Health Readiness assessment that integrates a person’s goals, digital health literacy, relevant electronic health record data, and key skill competencies needed to achieve those goals may be helpful for clinicians to integrate into their workflows [50]. Targeted education to improve technical literacy among older adults may also be necessary to equitably reach older populations. Medical complexity as a barrier, potentially distinct from age itself, aligns with other work examining barriers to video use among high-risk, high-need patients [51], underscoring the need to better understand how specific medical conditions may influence patients’ ability to engage in in-home video visits. However, age was a stand-alone barrier for some, which suggests ageist bias may limit clinician’s offer of video, a concern noted elsewhere [19]. Encouraging clinicians to examine their own attitudes toward older adults through tools such as the World Health Organization Ageism Scale [52] may help diminish the impact of ageist attitudes.

Our observation regarding the importance of caregivers to in-home video visits, especially for older patients, aligns with work relaying older adults’ frequent reliance on family members to help with technology [53]. Related to caregiver support for specific types of care, our own work demonstrates the centrality of caregivers for occupational therapy video visits [54,55]. Caregivers are particularly important for dynamic telehealth, such as synchronous home safety assessments, in which remote clinicians conduct an in-home assessment while the caregiver walks around holding a portable computing device such as a laptop, tablet, or smartphone [40,56,57]. Caregivers are also integral for in-home video visits to optimize older patients’ posthospital mobility and physical activity [58] and for dementia management [59]. While clinicians may ask patients who are hesitant to try in-home video about seeking help from caregivers, it is important to acknowledge potential barriers. Caregivers may have their own technical and functional challenges that affect their ability to support patient engagement in video visits and may vary in their preferences. A recent study of caregivers of homebound patients found that caregivers had a range of perspectives about the utility of video, while acknowledging its benefits for their loved one [60]. In another study examining caregivers’ perspectives of the role of technology in dementia care, caregivers noted that lack of resources, such as the cost of technology, is a barrier [61]. The need to provide resources to patients to engage is also echoed by clinicians [38], which extends to caregivers as well. Some patients may also lack a caregiver, necessitating the development of innovative alternatives that can provide one-to-one technical assistance, such as Video Visits for Elders Project [62].

### Geographical and Health System Factors

The impact of patient distance from the medical center on whether video is offered aligns with evidence for telehealth increasing access to care [63], suggesting that clinicians consider offering in-home video as an alternative for patients who live far from their medical center. However, our findings broaden access challenges to include patients with mobility and other impairments for whom attending in-person clinic visits from any distance is challenging. This accords with our prior study of caregivers of patients with dementia who reported that mobility and the impact of cognitive impairment (in the form of behavioral symptoms of dementia) create logistical challenges from any distance [64,65]. This suggests that clinicians consider in-home video as an option for older patients with medical complexities or mobility challenges, regardless of their proximity to care. Finally, VA’s telehealth infrastructure, which provides software, tablets, and technical support, is conducive to offering in-home video, whereas the absence of similar hardware provisioning outside VA is a barrier [66]. This underscores the need to address telehealth resource shortages through tablet loaner programs outside the VA [67]. It also highlights the need to address persistent challenges to in-home video in rural areas—which is where many older veteran patients reside—such as lack of high-speed internet connectivity [68]. Taken together, addressing TechQuity in in-home video telehealth for older adults requires a comprehensive approach.

### Limitations and Future Directions

This study has several limitations. First, our findings regarding factors underlying clinicians’ offer of video telehealth may not generalize beyond VA, given the uniqueness of the veteran population and the VA’s substantial telehealth infrastructure. Second, this was a convenience sample in which all participants had experience using video telehealth with older adults. Therefore, their perspectives may not generalize to clinicians without such prior experience. Relatedly, clinicians may have reported predominantly about factors influencing their offer as long-term users of video telehealth rather than factors affecting novice users, which may limit implications for clinicians with less or little experience with video telehealth. We also did not gather clinician participant demographic factors, which also limits generalizability. Selection bias is a potential limitation, as all participants were identified by our team or known colleagues. Finally, we did not examine the relative weight of influential factors to determine which, if any, are most influential to clinician decision-making. Future work should examine the differential influence of varied factors on clinicians’ offer of in-home video to older adults, as well as potential interactions between factors, in large-scale studies.

## Appendix

Multimedia Appendix 1 Planning for and Assessing Rigor in Rapid Qualitative Analysis (PARRQA) 18-recommendation checklist.

## Abbreviations

HIPAA: Health Insurance Portability and Accountability Act
RREAL: Rapid Research, Evaluation and Appraisal Lab
VA: Veterans Affairs

## Data Availability

The deidentified consensus Rapid Research, Evaluation and Appraisal Lab sheet data that support the findings of this study are available from the corresponding author upon reasonable request.
