# Supplementary material for: Clinician Decision-Making Around Offering Home Video Telehealth Visits: Qualitative Study
Source: JMIR Form Res. 2026 Jun 18;10:e83800. doi: 10.2196/83800 (PMC13329330; doi:10.2196/83800)
Supplement: Multimedia Appendix 1 [file formative_v10i1e83800_app1.pdf]

PARRQA 18-Recommendation Checklist

Item	Recommendation Guidelines	Page
1.	Clarify primary evaluation or research questions.	3
2.	Document rationale for using rapid qualitative approach.	3-5
3.	Specify planned rapid qualitative method and data sources.	4-5
4.	Identify guiding theories, frameworks, or models.	4
5.	Outline a realistic timeline for phases and deliverables.	3-4
6.	Confirm staffing, roles, and time commitments.	3-4
7.	Develop focused interview or observation guide.	4
8.	Pilot test guide and refine based on feedback.	4
9.	Define sampling approach, criteria, and recruitment strategy.	4
10.	Plan consistent and secure data capture methods.	4
11.	Establish clear field procedures and documentation processes.	4
12.	Create summary template aligned with domains.	4-5
13.	Calibrate team for consistent summarizing.	5
14.	Include accuracy checks against source data.	4
15.	Use version control for summary templates.	5
16.	Design analytic matrices for cases/themes.	5
17.	Define rules for populating matrices.	5
18.	Conduct cross-case comparisons for patterns.	5

Cite: Kowalski CP, Nevedal AL, Finley EP, Young JP, Lewinski AA, Midboe AM, Hamilton AB. Planning for and Assessing Rigor in Rapid Qualitative Analysis (PARRQA): a consensus-based framework for designing, conducting, and reporting. Implement Sci. 2024 Oct 11;19(1):71. doi: 10.1186/s13012-024-01397-1. PMID: 39394597; PMCID: PMC11468362.
